# Genomic Variation and Arsenic Tolerance Emerged as Niche Specific Adaptations by Different *Exiguobacterium* Strains Isolated From the Extreme Salar de Huasco Environment in Chilean – Altiplano

**DOI:** 10.3389/fmicb.2020.01632

**Published:** 2020-07-15

**Authors:** Juan Castro-Severyn, Coral Pardo-Esté, Katterinne N. Mendez, Naiyulin Morales, Sebastián L. Marquez, Franck Molina, Francisco Remonsellez, Eduardo Castro-Nallar, Claudia P. Saavedra

**Affiliations:** ^1^Laboratorio de Microbiología Aplicada y Extremófilos, Facultad de Ingeniería y Ciencias Geológicas, Universidad Católica del Norte, Antofagasta, Chile; ^2^Laboratorio de Microbiología Molecular, Facultad de Ciencias de la Vida, Universidad Andres Bello, Santiago, Chile; ^3^Center for Bioinformatics and Integrative Biology, Facultad de Ciencias de la Vida, Universidad Andres Bello, Santiago, Chile; ^4^Sys2Diag CNRS/Alcediag, CNRS UMR 3145, Montpellier, France; ^5^Centro de Investigación Tecnológica del Agua en el Desierto-CEITSAZA, Universidad Católica del Norte, Antofagasta, Chile

**Keywords:** *Exiguobacterium* genus, genomics, arsenic, niche, poly-extremophilic

## Abstract

Polyextremophilic bacteria can thrive in environments with multiple stressors such as the Salar de Huasco (SH). Microbial communities in SH are exposed to low atmospheric pressure, high UV radiation, wide temperature ranges, salinity gradient and the presence of toxic compounds such as arsenic (As). In this work we focus on arsenic stress as one of the main adverse factors in SH and bacteria that belong to the *Exiguobacterium* genus due to their plasticity and ubiquity. Therefore, our aim was to shed light on the effect of niche conditions pressure (particularly arsenic), on the adaptation and divergence (at genotypic and phenotypic levels) of *Exiguobacterium* strains from five different SH sites. Also, to capture greater diversity in this genus, we use as outgroup five As(III) sensitive strains isolated from Easter Island (Chile) and The Great Salt Lake (United States). For this, samples were obtained from five different SH sites under an arsenic gradient (9 to 321 mg/kg: sediment) and isolated and sequenced the genomes of 14 *Exiguobacterium* strains, which had different arsenic tolerance levels. Then, we used comparative genomic analysis to assess the genomic divergence of these strains and their association with phenotypic differences such as arsenic tolerance levels and the ability to resist poly-stress. Phylogenetic analysis showed that SH strains share a common ancestor. Consequently, populations were separated and structured in different SH microenvironments, giving rise to multiple coexisting lineages. Hence, this genotypic variability is also evidenced by the COG (Clusters of Orthologous Groups) composition and the size of their accessory genomes. Interestingly, these observations correlate with physiological traits such as growth patterns, gene expression, and enzyme activity related to arsenic response and/or tolerance. Therefore, *Exiguobacterium* strains from SH are adapted to physiologically overcome the contrasting environmental conditions, like the arsenic present in their habitat.

## Introduction

*Exiguobacterium* is a bacterial genus initially described by Collins et al. (1983), as Gram positive, pigmented rods, facultative aerobic with a wide range of temperature and tolerance. These bacteria have also been characterized as halo-, psychro- and thermo-tolerant and some even resist highly toxic arsenic (Vishnivetskaya et al., 2007; Belfiore et al., 2013; Remonsellez et al., 2018; Castro-Severyn et al., 2019). Different strains from this genus have been isolated from multiple extreme environments that vary from marine water, permafrost, deserts, salt flats and even stromatolites (Rodrigues et al., 2006; Ordoñez et al., 2013; Tang et al., 2013; Vishnivetskaya et al., 2014; Zhang et al., 2015). The presence of these bacteria in various environments and under different extreme conditions is evidence of their great plasticity and adaptability, which correlates with the great genetic diversity shown (Vishnivetskaya et al., 2009; Castro-Severyn et al., 2017; da Costa et al., 2018).

The Salar de Huasco is a poly-extreme ecosystem located on the Chilean Altiplano (Northern Region) where cultivable members of the *Exiguobacterium* genus are ubiquitous (Remonsellez et al., 2018). This area is subjected to major climatic oscillations (Cortés-Albayay et al., 2019), namely: temperature, salinity, high levels of solar radiation, negative water balance and the presence of arsenic (Dorador et al., 2008; Herrera et al., 2009; Hernández et al., 2016; Pérez et al., 2017; Remonsellez et al., 2018; Castro-Severyn et al., 2019). Another relevant aspect of the Salar de Huasco is the great microbial diversity that has been little explored (Dorador et al., 2010, 2013; Molina et al., 2016; Castro-Severyn et al., 2017). The co-occurrence of stress factors throughout the whole Chilean Altiplano and the great diversity of microorganisms able to prosper under these extreme conditions have promoted these ecosystems as models for extra planetary life to test the limits of life, as well as reservoirs for new/unknown metabolism pathways/molecular mechanism (Cabrol et al., 2009a, b, 2018).

Arsenic is a toxic metalloid mainly found as arsenate: As(V) or arsenite: As(III) in the environment, which easily enters to the cells causing severe damages (Xiao et al., 2016). Hence, As(V) replaces phosphate inhibiting all the reactions in which this participates and As(III) reacts with thiol groups, inhibiting enzymes function and promoting the generation Reactive Oxygen Species (ROS), thus interfering with the cell redox state maintenance (Slyemi and Bonnefoy, 2012). The main arsenic tolerance mechanism (for both arsenic species) used by *Exiguobacterium* is the expulsion from the cell (Ordoñez et al., 2015; Castro-Severyn et al., 2017). Since, As(III) has several ways to be expelled from the cell, As(V) is reduced to As(III) to be linked and detoxified through the same mechanism (Andres and Bertin, 2016). Also, the main difference is that As(III) generates a much higher toxicity, therefore a cell tolerates this species better if has a broader response repertoire to quickly expel the toxic, repair damage and restore redox imbalance (Mateos et al., 2017).

In recent years, genomic analyses have aided to determine the degree of genetic divergence and the evolutionary origin of interesting adaptability markers. On the other hand, these approaches also allowed the discovery of new bacterial species and proposed the regrouping or separation of those previously classified (Whitman, 2015; Chun et al., 2018; Pérez-Losada et al., 2018). The reevaluation of the methodologies for bacterial classification is a current problem, especially when databases are being enriched with new sequences of unknown microorganisms that cannot be cultivated under laboratory conditions or studied under classical methodologies (Rosselló-Móra and Amann, 2015; Thompson et al., 2015). Considering arsenic metabolism, new markers related to its resistance have been identified, demonstrating that the classic/known mechanisms are not as rigid as initially proposed. Furthermore, these can coexist and be interconnected by accessory proteins depending on the bacteria and probably their surroundings (Zhao, 2016; Chang et al., 2018; Fekih et al., 2018; Shi et al., 2018).

Previously, we carried out a study of three *Exiguobacterium* strains focusing on the high levels of arsenic tolerance presented by isolates from the Salar de Huasco sediments. Through sequencing, genomic and proteomic analyzes of these strains, we found several markers directly related tolerance arsenic, as well as the response to oxidative and overall stress (Castro-Severyn et al., 2017, 2019; Remonsellez et al., 2018). In this study, we tested whether genotypic variability of *Exiguobacterium* isolates correlates with their capacity to tolerate arsenic and with specific properties of their habitat, which could drive genus divergence. Furthermore, these findings were compared with an outgroup of five *Exiguobacterium* strains isolated from Easter Island – Chile and the Great Salt Lake – United States environments.

## Materials and Methods

### Study Area and Sampling

In January of 2017 during a field work in the Salar de Huasco National Park (Chilean Altiplano), water and sediment samples were taken from five different sites (previously described by Dorador et al., 2008). The Salar area is known for its heterogeneity, considering spatially as well as biodiversity and physicochemical characteristics. This ecosystem is mostly composed of streams, salt crusts, peatlands and shallow (permanent and non-permanent) lakes with a salinity gradient from north to south (H0 to H5; Dorador et al., 2008). Physicochemical parameters like temperature, salinity and pH were recorded *in situ* (HI 98192 and HI 2211 - HANNA Instruments). Total arsenic concentration was determined by the INQUISAL-CONICET service (San Luis, Argentina), through an ELAN DRC-e ICP-MS (PerkinElmer^®^) following the ASTM “American Society for Testing and Materials” standard methods (TMECC: 04.12-B and 04.14).

### Culture, Isolation and Identification of *Exiguobacterium* Strains

All samples were inoculated for enrichment and isolation of halophilic/halotolerant bacteria into YP medium (2 g/l yeast extract, 5 g/l Peptone, 25 g/l NaCl and pH 7.8) supplemented with 1 mM of NaAsO_2_ [As(III)] and incubated at 25°C for 24 h only for culture enrichments. Then, bacteria were then plated in the same medium (including 12 g/l of agar) to isolate different bacterial morphotypes by dilution method. The plates were incubated at 25°C until the appearance of colonies. We set up to pick the characteristic orange pigmented colonies and also through microscopy we selected the Gram-positive rods. Besides, we also included seven other previously isolated strains, five from Easter island (Chile) (Cumsille et al., 2017), which were provided by Dra. Beatriz Cámara from Universidad Técnica Federico Santa María (Valparaíso, Chile). Along with, two more strains isolated from the north shore of the Great Salt Lake (Utah – United States) sediment, which were supplied by David Garcia from Brigham Young University (Hawaii – United States). Taxonomic classification for all the strains was carried out by 16S rRNA amplicon sequencing using the 27F and 1525R primers (Rainey et al., 1996) through the Sanger method (ABI PRISM 3500xl Applied Biosystems – Centro de Secuenciación Automática de ADN, Pontificia Universidad Católica de Chile). For classification, sequences were assembled and queried against GenBank (Benson et al., 2000) and SILVA v132 (Quast et al., 2012) 16S rRNA databases.

### Arsenic Tolerance

Minimal inhibitory concentration (MIC) assays for As(III) and As(V) were performed for all isolated *Exiguobacterium* strains. Briefly, bacterial cultures in Luria-Bertani broth (LB) were grown at 25°C with constant agitation (150 rpm) until 0.4 of OD_600_. Following, we set up a micro plate with dilutions of As(III): NaAsO_2_ and As(V): Na_3_AsO_4_ to final concentrations from 0.1 to 25 mM and 10 to 300 mM, respectively. Controls were made by adding fresh medium to the corresponding well instead of arsenic. Each well was inoculated with the grown culture in 1:20 ratio in LB medium. Finally, the plates were incubated at 25°C for 48 h with constant agitation, and OD_600_ values were read with a TECAN Infinite 200 PRO Nanoquant.

### Genomes Sequencing, Assembly and Annotation

Total DNA was extracted from each selected strain using the GeneJET Genomic DNA Purification Kit (Thermo Fisher Scientific) according to manufacturer’s instructions. DNA integrity, quality, and quantity were verified using 1% agarose gel electrophoresis, OD_260/280_ ratio and fluorescence using a Qubit^®^ 3.0 Fluorometer along with the Qubit dsDNA HS Assay Kit (Thermo Fisher Scientific). Then, the samples were sent to MicrobesNG (University of Birmingham, United Kingdom) for library construction and sequencing. Briefly, genomic DNA of each strain was used to construct paired-end (250 bp reads) libraries using the Nextera XT Library Prep Kit (Illumina^®^) following the manufacturer’s protocol and sequencing was performed through Illumina HiSeq platform. An average on 9.1 million reads per sample were obtained, representing an average depth of 78X. Output reads were adapter trimmed using Trimmomatic v0.30 (Bolger et al., 2014). Quality control was performed using FastQC v0.11.8 (Andrews, 2010) for evaluation and PRINSEQ v0.20.4 (Schmieder and Edwards, 2011) for filtering and trimming (thresholds: Ns = 0, read length ≥ 150 bp and Q ≥ 20). *De novo* assembly was carried out with SPAdes v3.7 (Bankevich et al., 2012), and resulting contigs were annotated with Prokka v1.13.3 (Seemann, 2014) and eggNOG-mapper v1.0.3 (Huerta-Cepas et al., 2017). Genome assemblies were evaluated by statistical values calculation with QUAST v5.0.2 (Gurevich et al., 2013) and completeness analysis through the search of bacterial ortholog genes (OrthoDB v9 database: Zdobnov et al., 2016), using BUSCO v3 (Waterhouse et al., 2017). The Whole Genome Shotgun Project (assemblies and biosamples) has been deposited at DDBJ/ENA/GenBank under the Bioproject: PRJNA319980.

### *Exiguobacterium* Genomic Data Sets

Three different genomic data sets were used for the following analyzes: first we included the genomes of the 14 strains isolated from the five Salar de Huasco sites, including the previously described SH31 strain (Castro-Severyn et al., 2017). In the second set, we included the five outgroup strains (three from Easter Island and two from the Great Salt Lake). Finally, for the third set we include all available *Exiguobacterium* genome sequences deposited in GenBank as of March 2019, for a total of 90 genomes. Among these there were 23 MAGs (Metagenome assembled genomes) of which, 16 were left out because of completeness problems (< 90%), resulting on 74 genomes total (Supplementary Table S1). All the genome assemblies were re-annotated using Prokka v1.10 (Seemann, 2014), to make them comparable.

### Phylogenetic Relationships

Thirty-one phylogenetic gene markers (implemented in AMPHORA, Wu and Eisen, 2008), were extracted from each genome in both data sets. All nucleotide sequences were translation aligned using MAFFT (Katoh and Standley, 2013) as implemented in Geneious^®^ v7.1.9 software (Kearse et al., 2012). The alignments were then concatenated using Seqotron v1.0.1 (Fourment and Holmes, 2016). The best partitioning scheme was identified, using the program PartitionFinder v2.1.1 (Lanfear et al., 2016). A distribution of probable trees was obtained by Bayesian Inference with MrBayes v3.2.6 (Ronquist et al., 2012). Two separate runs of 20 million generations were executed (four chains each run; sampling every 1,000 generations). The resulting tree was visualized using FigTree v1.4.4^1^. The average nucleotide identity (ANI) was calculated using the pyANI Python3 module (Pritchard et al., 2019) and average amino-acid identity (AAI) was calculated using the CompareM toolkit^2^. R package pheatmap was used for results visualization (Kolde, 2015).

### Pan Genome Analysis

Pan genome was defined clustering the proteins families into ortholog groups based on their sequence similarity using the algorithm orthoMCL v1.4 (Li et al., 2003) as implemented in GET_HOMOLOGUES (Contreras-Moreira and Vinuesa, 2013). This analysis was carried out with the 14 Salar de Huasco genomes and the core genome is defined by the protein clusters that are present in ≥ 13 of the 14 genomes. On the other hand, accessory genome is defined by the protein clusters that are present in ≤ 2 of the 14 genomes, leaving all those clusters present in 3 to 12 genomes on the disposable genome category. To highlight the differences between the strain’s accessory genomes, composition of COG (Clusters of Orthologous Groups) categories was analyzed and compared. All visualizations were generated in the ggplot2 R package (Wickham, 2016).

### Protein Searches

Bi-directional best hit searches using Blast (Altschul et al., 1990) were performed to infer homology between reference proteins from UniProt (UniProt Consortium, 2019) and the predicted genes from the *Exiguobacterium* datasets (a minimum e-value of 1E^–05^ and filters of 80% for query coverage and 70% for identity were applied). This strategy was used to identify and compare arsenic tolerance and metabolism proteins and those involved with stress-response. Genetic context was visualized using Geneious^®^ v7.1.9 software (Kearse et al., 2012) and protein functions were verified using several tools as Pfam (El-Gebali et al., 2019), GOFeat (Araujo et al., 2018) and InterPro (Jones et al., 2014); also, String v11.0 (Szklarczyk et al., 2014) was used for co-occurrence and neighborhood analyzes.

### Effect of Arsenic on Growth

Growth kinetics selected *Exiguobacterium* strains under different As(III) and As(V) concentrations (Supplementary Table S2) were monitored for 24 h at 25°C with continuous orbital agitation (150 rpm). OD_600_ measures were recorded every hour. The control condition was equally prepared but without the addition of arsenic. Each assayed condition was performed in three independent experiments with three technical replicates each and visualized using R package ggplot2 (Wickham, 2016).

### Biochemical Reactive Oxygen Species Indicators and Antioxidant Activity

Since oxidative stress has been described as a consequence of arsenic toxicity, we measured on the three selected strains some indicators of this process. For this, bacteria were grown on LB medium at 25°C with continuous orbital agitation (150 rpm), up to 0.4 of OD_600_. Three different experimental conditions (Control without arsenic and half of strain specific MIC for As(III) and As(V) were tested; Supplementary Table S2). The intracellular accumulation of reactive oxygen species (ROS) was determined using 10 μM of the fluorescent probe 2′,7′-dichlorodihydrofluorescein diacetate (H_2_DCFDA), as described by Echave et al. (2003). Briefly, bacteria were washed and re-suspended in Tris buffer (Tris–HCl 50 mM, pH 7.8) and fluorescent probe was added just prior measuring, and fluorescence (excitation 480 nm; emission 520 nm) was recorded on a microplate reader (Infinite^®^ 200 Pro, Tecan) every 5 min for a total period of 100 min. For the calculation, emission values for each measurement point were first blanked against the background fluorescence of bacteria without the probe and then normalized with the corresponding (blanked) OD_600_ of bacteria measured at the same time as the fluorescence was read. The difference in fluorescence was calculated and divided by the elapsed time, this value was normalized by the difference in growth during the respective time.

For enzymatic assays, protein extracts were obtained by sonic disruption of bacteria grown under the same conditions described above. Bacterial cells were harvested by centrifugation (3,000 *g*, 10 min), washed twice with Tris buffer and pellets were re-suspended in 1 ml of the same buffer supplemented with 1 mM of PMSF. Sonication was carried out with 40% amplitude, 130 watts, 20 kHz, during 5 min (10 s on and 10 s off cycles) in an Ultrasonic Processor VCX-130 (Sonics, Inc.). The lysates were centrifuged at 24,000 *g* for 40 min at 4°C to recover the supernatants and protein concentration was measured using the Coomassie (Bradford) Protein Assay (Thermo Scientific).

Catalase activity was determined spectrophotometrically following the protocol previously described by Chen et al. (2003). Briefly, a solution with 20 mM of H_2_O_2_ in 250 mL of Tris buffer was prepared, followed by the addition of 10 μL of protein extract in a 96 well UV-plate, the H_2_O_2_ hydrolysis was measured and monitored at 240 nm every 30 s for 3 min, using a microplate reader (Infinite^®^ 200 Pro, Tecan). Superoxide dismutase (SOD) activity was assessed by measuring the inhibition of the photochemical reduction of nitro blue tretrazolium (NBT) from the protein extracts previously described (Jakubowski et al., 2000; Guerrero et al., 2013). OD_550_ was measured after 15 min illumination. A SOD unit was defined as the amount of enzyme causing 50% inhibition of NBT reduction.

All the assays were performed in at least three independent experiments with three technical replicates each. One-way ANOVA with post hoc Tukey HSD test was used for all comparisons and a *P*-value < 0.05 was considered statistically significant. All the statistics were performed using GraphPad Prism v5.0 (Prism^®^) and visualizations were made using R package ggplot2 (Wickham, 2016).

### Transcriptional Response to Arsenic

The relative expression/transcripts levels of genes involved direct and indirectly on arsenic tolerance were quantified by RT-qPCR. Selected strains were grown in the same previously mentioned conditions (control without arsenic, half of specific MIC, for both As(III) and As(V): Supplementary Table S2). After, cultures were pelleted, and RNA extractions were carried out using the GeneJET RNA Purification Kit (Thermo Fisher Scientific) according to manufacturer’s instructions. RNA integrity, quality, and quantity were verified using 1% agarose electrophoresis, OD_260/280_ ratio and the QuantiFluor RNA System (Promega^®^). cDNA was synthesized using the M-MLV Reverse Transcriptase kit (Promega^®^) and Random Primer oligonucleotides hexamers (Invitrogen^TM^). The PCR reaction was carried out as follows: 10 min at 95°C followed by 40 amplification cycles (95°C × 30 s, 58°C × 30 s, 72°C × 30 s), and a final step of 95°C × 15 s; 25°C × 1 s; 70°C × 15 s and 95°C × 1 s) using specific primers for each gene (Supplementary Table S3). Transcript levels were quantified using the Brilliant II SYBR Green qPCR Master mix kit (Agilent Technologies) on a Stratagene Mx3000P thermal cycler (Agilent Technologies). Gene expression levels were calculated according to Pfaffl (2001) using 16S rRNA gene as normalizator. One-way ANOVA with post hoc Tukey HSD test was used for all comparisons and a *P*-value < 0.05 was considered statistically significant (GraphPad 5.0: Prism^®^) and visualizations were made using R package ggplot2 (Wickham, 2016).

## Results

### Salar de Huasco (SH) Sampling and Environmental Characteristics

**FIGURE 1:**
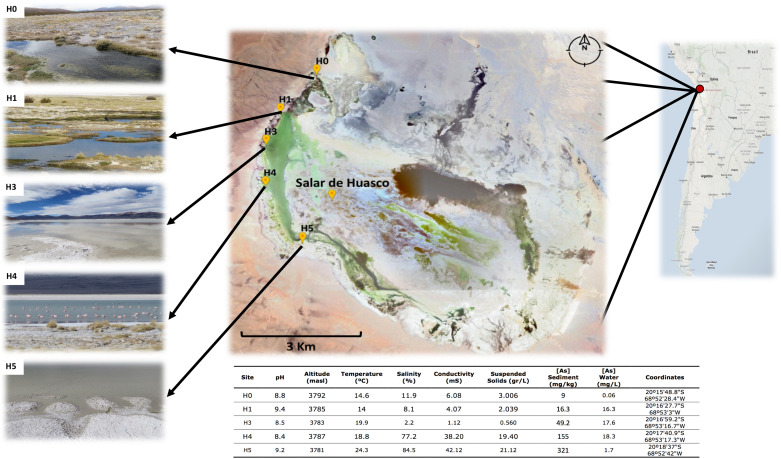
Salar de Huasco sampling sites and characteristics. Above: Salar de Huasco localization and map indicating the five sampled sites from which the studied strains were isolated. Source: Google-Earth. Below: table of the five sites environmental and physicochemical measured characteristics.

The spatial variation between the five sampled sites span a distance of 5.9 km (Figure 1: Map), in particular the distances between sites are: H0-H1: 1.59; H1-H3: 1.03; H3-H4: 1.31 and H4-H5: 2.01 Km. The environmental variables recorded (Figure 1 and Table 1) presented wide range of variations, in particular arsenic concentration in sediment (9 – 321 mg/kg), salinity (2.2 – 84.5 %), conductivity (1.1 – 42.1 mS) and suspended soils (0.56 – 21.12 g/L). As it has been reported before, salinity increases in a gradient from north to south (Dorador et al., 2010). It also appears to be the case for arsenic concentration in sediments, which was reported recently for the first time by our group in three of these five sites (Castro-Severyn et al., 2019). Here we added two more sites, which fit perfectly with the proposed gradient.

**TABLE 1:** Identification of all selected isolates through 16S rRNA sequencing.

**Site**	**Strain**	**% Identity**	**% Query Cover**	**BLAST Hit**
SH-H0	SH0S2	95	99	*Exiguobacterium* sp. AC-CS-C2
	SH0S3	96	99	*Exiguobacterium* sp. AC-CS-C2
	SH0S7	98	99	*Exiguobacterium* sp. AC-CS-C2
	SH0S1	99	98	*Exiguobacterium* sp. AC-CS-C2
SH-H1	SH1S4	98	95	*Exiguobacterium* sp. AC-CS-C2
	SH1S1	99	97	*Exiguobacterium* sp. AC-CS-C2
	SH1S21	98	97	*Exiguobacterium* sp. SH31
SH-H3	SH3S1	99	98	*Exiguobacterium* sp. AC-CS-C2
	SH3S2	96	98	*Exiguobacterium* sp. AC-CS-C2
	SH3S3	99	97	*Exiguobacterium* sp. AC-CS-C2
SH-H4	SH4S7	98	98	*Exiguobacterium* sp. SH31
	*SH31	**99**	**99**	***Exiguobacterium* sp. AC-CS-C2**
SH-H5	SH5S7	95	98	*Exiguobacterium* sp. AC-CS-C2
	SH5S13	96	99	*Exiguobacterium* sp. SH31
	SH5S32	98	98	*Exiguobacterium mexicanum* HUD
	SH5S4	99	99	*Exiguobacterium* sp. AC-CS-C2
	SH5S20	98	99	*Exiguobacterium* sp. AC-CS-C2
GSL	SL-9	95	99	*Exiguobacterium* sp. BAB-5887
	SL-10	95	98	*Exiguobacterium* sp. BAB-5887
EI	IPBC4	98	98	*Exiguobacterium aurantiacum* Q3-11
	IPCI3	96	98	*Exiguobacterium aurantiacum* Q3-11
	IPCH1	99	99	*Exiguobacterium aurantiacum* 104NE
	IPBC7	95	99	*Exiguobacterium aurantiacum* 104NE
	IPCG2	97	97	*Exiguobacterium aurantiacum* Q3-11

SH, Salar de Huasco sites, Chile; GSL, Great Salt Lake; Salt Lake City – Utah, United States; EI, Easter Island, Motu Nui Islet, Chile. *Previously reported strain (Castro-Severyn et al., 2017).

### Bacterial Culture, Isolation and *Exiguobacterium* Identification

Different bacterial morphotypes were isolated from sample enriched cultures and 16S rRNA molecular identification yielded 16 new *Exiguobacterium* strains from the SH sites. The taxonomic affiliation of the other 7 outgroup strains (5 from Easter Island and 2 from the Great Salt Lake) was also molecularly checked. All the rRNA 16S sequences were classified with confidence values for identity (≥ 95%) and coverage (≥ 95%) (Table 1). It should be noted that the strains from the three environments showed similarity with different *Exiguobacterium* strains from the databases.

### Arsenic Tolerance and Selected Strains Genome Sequences

Arsenic tolerance levels among all the strains showed great diversity, especially for As(III) in the SH strains. This could be due to the contrasting arsenic concentrations found in each environment (Table 2). Particularly, As(V) tolerance was relatively homogeneous spanning from 100 to 200 mM. Moreover, As(III) tolerance was very heterogeneous spanning from 1 to 20 mM among the strains. Interestingly, the distribution of the tolerance levels among the strains do not follow the arsenic gradient, not a logical organization by site. However, the three strains from the H1 site were those who showed the lowest tolerance to As(III). On the other hand, EI strains tolerance was comparable to the SH ones for As(V) but displayed complete sensitivity for As(III). While, GSL isolates showed complete sensibility for As(III) and a very low for As(V).

**TABLE 2:** Minimal inhibitory concentration of both As species for all the isolated strains; Accession number and assembly evaluation of the selected strains genomes.

**Site**	**Strain**	**[mM] As(III)**	**[mM] As(V)**	**Selected for sequencing**					
				**GenBank assembly**	**Size (mb)**	**GC %**	**# contigs**	**N50**	**% completitude**
SH-H0	SH0S2	10	200	GCA_004337185.1	2.94	52.00	18	758317	100
	SH0S3	2.5	200						
	SH0S7	20	150	GCA_004337195.1	2.93	51.80	35	256028	100
	SH0S1	7.5	200	GCA_004337165.1	2.91	51.70	18	474352	100
SH-H1	SH1S4	1	200	GCA_004337095.1	2.91	52.10	22	439798	100
	SH1S1	1	200	GCA_004337245.1	2.91	52.10	24	257075	100
	SH1S21	1	150	GCA_004337175.1	2.92	51.80	27	236663	100
SH-H3	SH3S1	15	200	GCA_004337105.1	2.76	52.20	32	214817	100
	SH3S2	2.5	200	GCA_004337285.1	2.70	52.20	36	202019	100
	SH3S3	7.5	200	GCA_004337115.1	2.69	52.20	33	202019	100
SH-H4	SH4S7	10	200	GCA_004336795.1	2.94	52.00	23	428140	100
	*SH31	**10**	**100**	**GCA_001816105.1**	**3.02**	**51.70**	**120**	**44068**	**98**
SH-H5	SH5S7	10	200						
	SH5S13	15	100	GCA_004337085.1	2.92	51.90	74	84368	100
	SH5S32	15	100	GCA_004336775.1	2.91	52.10	24	257075	100
	SH5S4	15	200	GCA_004337045.1	3.05	51.60	64	83702	100
	SH5S20	10	200						
GSL	SL-9	0	10	GCA_004336985.1	2.98	51.30	15	815925	100
	SL-10	0	10	GCA_004337025.1	2.93	51.50	33	216935	100
EI	IPBC4	0	100	GCA_004337065.1	2.97	52.00	39	156917	99.4
	IPCI3	0	100	GCA_004337275.1	2.97	52.00	38	156123	100
	IPCH1	0	100	GCA_004337295.1	2.97	52.00	40	156123	99.3
	IPBC7	0	100						
	IPCG2	0	100						

SH, Salar de Huasco sites; GSL, Great Salt Lake; EI, Easter Island. * Previously reported strain (Castro-Severyn et al., 2017).

From the complete strains set, 18 were selected for whole genome sequencing (including 13 of our SH isolates), according to their origin and arsenic tolerance, trying to cover the whole set diversity. Hence, the resulting assembled genomes yielded high quality values, meaning N50 as proxie for genome fragmentation level and the completeness percentage (≥ 99%). Furthermore, other features such as contigs number, size, GC% and predicted open reading frames spans between 15–74, 2.69–3.05 Mb, 51.30–52.20% and 2775–3179, respectively (Table 2). Showing the important degree of genotypic diversity existing among the strains.

### Genomic Relationships

Phylogeny, ANI and AAI were analyzed for all the sequenced genomes set and also within the complete set of 74 strains to consider their placement among the whole available diversity. The phylogenetic tree of the 74 strains display the same previously reported pattern as they were separated into two big groups (Supplementary Figure S1; Castro-Severyn et al., 2017). Our 19 strains belong to the group II being coherent with their isolation environments, as it was reported before which include the strains from marine, saline, temperate and alkaline environments (Vishnivetskaya et al., 2009).

All the SH strains appear to be monophyletic and those from EI and GSL belong to different clades. Notably, the *E. aurantiacum* PN47 strain is included in this branch, as it was isolated from sediment of another SH site (at 3.15 km south-east from H5 site), which explains why it is within our group (Strahsburger et al., 2018). Hence, our results suggest that the *E. aurantiacum* PN47 strain is nor correctly classified, being more related to those isolates from the SH. Thus, is much closer to our SH strains, regarding the *aurantiacum* species (ANI: 82.8% against other *aurantiacum* vs. 98.4 – 98.6% against de SH strains; AAI: 87.4% against other *aurantiacum* vs. 97.2 – 98.6% against the SH strains) (Supplementary Figures S2, S3).

**FIGURE 2:**
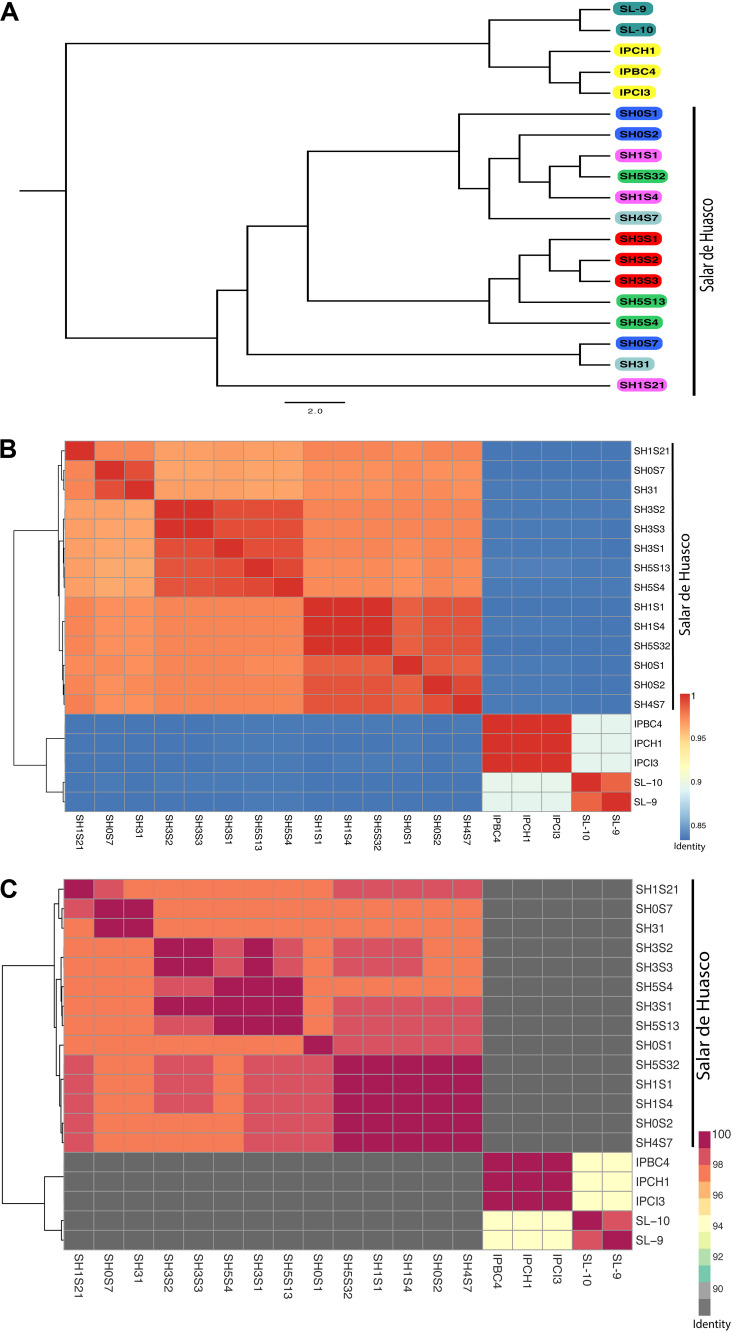
Genomic relationships among *Exiguobacterium* strains. **(A)** Mid-point rooted Phylogenetic tree inferred from an alignment of 31 conserved genes. Strains in the tree are color coded by isolation site (Great Salt Lake are turquoise; Easter Island are yellow and Salar de Huasco: H0 site - blue; H1 site - pink, H3 site - red, H4 site - gray and H5 site - green. **(B)** Average nucleotide identity (ANI) heatmap. **(C)** Average amino-acid identity (AAI) heatmap. Hierarchical clustering of the *Exiguobacterium* genomes based on their average nucleotide/amino-acid identity values. The color gradients show the percentage of identity, from lowest to highest, that each pair of genomes shares.

Analyzing separately the clade containing the 14 SH genomes, we observed that there is an important degree of diversity (Figure 2A). The phylogenetic relationships do not reflect the strains isolation origin nor arsenic tolerance level. The whole genome nucleotide identity of all the 74 genomes display the same clustering showed by the phylogenetic tree with ANI values that range from 72% to 99.999% (Supplementary Figure S2). The low values are mostly intergroup and thus support their separation. Considering only the SH strains the ANI values are > 96% which imply a high level of similarity (Figure 2B). As previously discussed, we determined that the clusters are not grouped by origin or arsenic tolerance. Interestingly, SH31, SH0S7 and SH1S21 strains do group together although having different origins and they cover the broadest arsenic tolerance levels. The AAI analysis replicates precisely the results and topology observed in the ANI and phylogenetic analysis. Moreover, the identity values within the 74 genomes set ranked from 62.7%. Notably, this value is lower than the one presented by the ANI (72%) which goes against what would be expected since the AAI considers only proteins which should be more conserved (Supplementary Figure S3). This could be due to the great divergence shown between the two groups, whereas, within the SH set the values were much higher (> 96,8%) corresponding to strain similarity (Figure 2C).

### Huasco Pan-Genome

**FIGURE 3:**
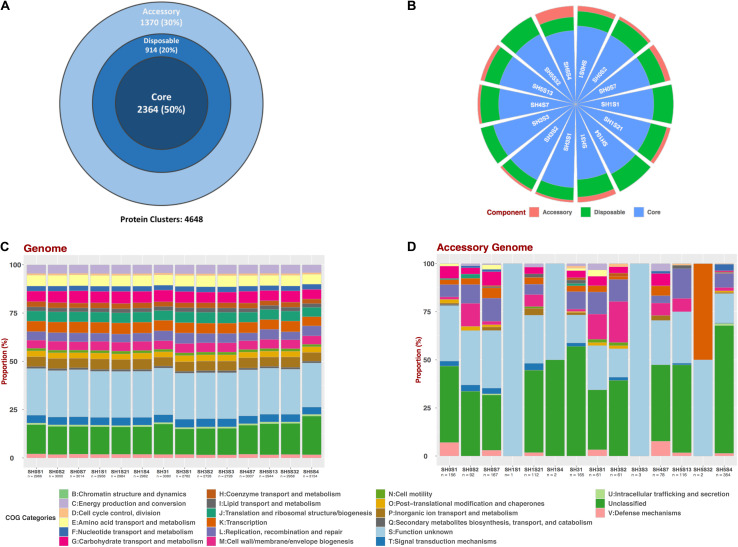
Pan-genomic analysis of SH *Exiguobacterium* genomes set. **(A)** Pan-genome compartments proportion representation for the whole set. **(B)** Compartments proportion for each genome. **(C)** Composition of COG categories for each whole genome and **(D)** For the accessory compartment.

To determine the genotypic diversity among the SH strains as well as the differences that could be attributed to their specific niche we performed a Pan-genome analysis with only these 14 strains (Figure 3). This pan-genome is composed of 4,648 protein clusters, of which 2,364 represents the core compartment ranging from 74.95 to 86.66% (average of 80%) among the genomes and a 50% of the whole pan-genome (Figure 3A). Additionally, the size of accessory and disposable compartments was variable for each strain, with a representation ranging between 0.03 – 5.54% and 11.11 – 20.12 %, respectively (Figure 3B and Table 3). This variation is consistent with the observed phylogenetic placement of these strains, supporting the idea that SH isolates are diverse even in the face of common origin.

**TABLE 3:** Proteins Classification for each genome in the pangenome compartments.

**Strain**	**Number of proteins**				**Compartment (%)**		
	**Total**	**Accessory**	**Disposable**	**Core**	**Accessory**	**Disposable**	**Core**
SH0S1	2966	156	446	2364	5.26	15.04	79.70
SH0S2	3000	92	544	2364	3.07	18.13	78.80
SH0S7	3014	167	483	2364	5.54	16.03	78.43
SH1S1	2958	1	593	2364	0.03	20.05	79.92
SH1S21	2984	112	508	2364	3.75	17.02	79.22
SH1S4	2962	2	596	2364	0.07	20.12	79.81
SH31	3080	165	551	2364	5.36	17.89	76.75
SH3S1	2782	61	357	2364	2.19	12.83	84.97
SH3S2	2728	61	303	2364	2.24	11.11	86.66
SH3S3	2728	3	361	2364	0.11	13.23	86.66
SH4S7	3007	78	565	2364	2.59	18.79	78.62
SH5S13	2944	116	464	2364	3.94	15.76	80.30
SH5S32	2958	2	592	2364	0.07	20.01	79.92
SH5S4	3154	354	436	2364	11.22	13.82	74.95

Moreover, to determine if this variability results in functional convergence or divergence, we performed a COG composition analysis. This was accomplished for all the predicted proteins (Figure 3C) and considering only those from the accessory compartment (Figure 3D) of each genome. The COG composition for the full set of proteins evidenced a homogeneous pattern between the strains. Moreover, slight variations in the proportions of some categories can be seen in SH31 and SH5S4 strains. On the other hand, accessory compartments are very different from each other, considering number of proteins, as well as COG composition. Unfortunately, most of the clusters in the accessory genome compartments are unclassified ones or with unknown function, thus more investigation is needed.

### Arsenic and Stress Tolerance Markers

**FIGURE 4:**
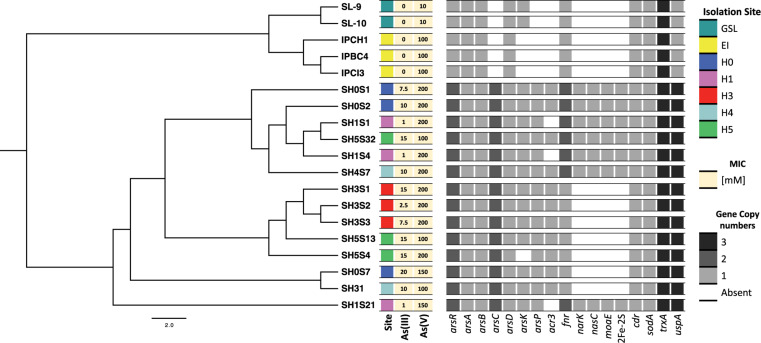
Identification of interest genes on *Exiguobacterium* genomes. Distribution and copy number of arsenic resistance genes among the genomes, organized by their phylogenetic relationships (as shown in Figure 2A). Heatmap shows color-coded genes copy numbers. Isolation sites correspond to the Great Salt Lake (GSL), Easter Island (EI) and the Salar de Huasco five sites (H0, H1, H3, H4 and H5).

The presence of arsenic and stress tolerance genes among the 19 *Exiguobacterium* genomes revealed that most of those related to global (*pdxS, dnaK, hpf, uspA, fur* and *luxS*) and oxidative stress (*cdr, katA, katE, sodA, gshAB, cysK, cysM, trxA, trxB, ydbP, ytpP, ahpF, bsaA, garB, tpx, bcp* and *resA*) were homogeneously present in all the strains (Supplementary Figure S4). However, there are differences regarding the copy number of genes such as *fnr* and *uspA*. Also, a pattern regarding isolation origin (environment) is evidenced in *uspA, dnaK* and *ydbP*. Regarding arsenic markers, *ars* operon genes were found in the 19 strains genomes. Interestingly, *acr3*, *arsC, arsP* and a second copy of *arsR* genes were exclusively detected in the SH (Figure 4). Moreover, the GSL it was the only environment where *arsK* gene was not detected. Even though the phylogenetic distribution does not correlate with the five SH sites nor with the arsenic resistance level presented by the strains. However, a group of clade specific genes (*narK, nasC, moaE* and a second copy of *fnr*) was detected.

**FIGURE 5:**
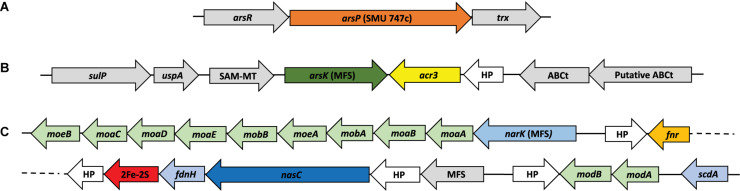
Genetic organization of Interest clusters found in *Exiguobacterium*. Show the genes context for those strains who have **(A)** *arsP*; **(B)** *arsK* – *acr3* and **(C)** *nasC* (as shown in Figure 4).

As it could have happened before, the miss-annotation of *arsP* caused it to be undetected in previous works. This could be a problem of database shortage, due the low identity percentage of this sequence with those available made unlikely to detect it. We used eggNOG, Pfam, InterPro and STRING (neighboring) tools to sum evidence and correctly annotate this protein (Figure 5A). The same strategy was used to identify *arsK*, which was recently reported to provide multi-resistance to different arsenic species like As(III), Rox(III), and MAs(III) (Shi et al., 2018). Here, we reported for the first time the presence of *arsK* for the *Exiguobacterium* genus. Notably, this gene is present in all but one SH strain (SH5S4) and on those from GSL. The genetic organization of *arsK* is neighboring with *acr3* efflux pump when both of them co-occurs (Figure 5B). Even though, *acr3* is only present in the SH strains with the exception of those isolated from the H1 site, which shockingly do not have it, which could account for their As(III) sensitivity (Table 2) (Ordoñez et al., 2015).

Another interesting exclusive feature found in half of SH strains, was the presence of what seems to be an assimilatory nitrate reductase (*nasC*) cluster of genes (Figure 5C), detected due to incorrect annotation a protein as an arsenite oxidase (*aioB*) which is actually a non-characterized 2Fe-2S protein that is within this gene cluster. There are also two MFS transporters, one uncharacterized and one that we annotated as the nitrate/nitrite transporter NarK. In addition, FdnH is a formate dehydrogenase that acts during anaerobic respiration, when nitrate is the electron acceptor (Kelley, 2017). This gene cluster had not been previously reported for *Exiguobacterium* genus and could be of high relevance in terms of physiological capabilities and niche adaptation that needs further investigation.

### Physiological Response to Arsenic

Aiming to cover the most diversity considering arsenic tolerance and geographic origin among the studied bacteria, we selected three strains for experimental analyzes. Specifically, the SH1S21, SH0S7 and SH31 strains were used for presenting the lowest, highest and mid-arsenic resistance, also for belonging from three different sites (Table 2). Additionally, it is of interest that the SH1S21 strain does not present the *acr3* gene.

Bacterial growth capacity was monitored for the three selected strains in the presence of different sublethal (up to half of the corresponding MIC value) As(V) and As(III) concentrations (Supplementary Figure S5). We found that all of them were able to grow under all tested conditions, as we reported before (Castro-Severyn et al., 2019). Notably, there was no significant difference between control and As(V) conditions, which has been reported previously (Ordoñez et al., 2015). On the other hand, As(III) causes a great effect on the growth pattern for the three strains, observing a delay in the time it takes to reach 0.4 of OD_600_ and a premature stationary phase. This is because the cell needs to alter its physiology to resist As(III) high toxicity, to maintain homeostasis and energy demand, thus the growth rate decreases (Cleiss-Arnold et al., 2010).

**FIGURE 6:**
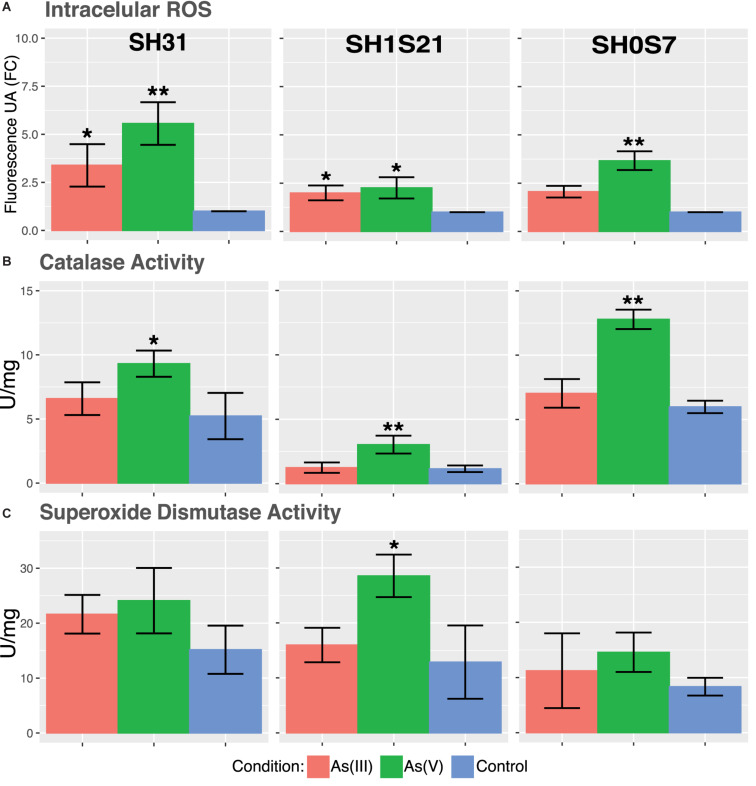
Physiologic response of some *Exiguobacterium* strains to arsenic stress. **(A)** Accumulation of intracellular ROS. **(B)** Catalase activity. **(C)** Superoxide dismutase activity. Data represents an average of three independent experiments with three technical replicates each (*p < 0.05; **p < 0.01; ***p < 0.001).

The accumulation of ROS are indicators of cellular stress and oxidative damage has been described as the main mechanism of arsenic toxicity, mainly due thiol depletion (Imlay, 2003; Slyemi and Bonnefoy, 2012). In this sense, ROS intracellular accumulation in the three strains show a significant increase when arsenic is present (Figure 6A). It can be noted that As(V) causes significantly higher ROS accumulation on the three strains, this could be related to the use of reduction mechanism to detoxify arsenic by the bacteria (Mukhopadhyay et al., 2002).

The mentioned ROS accumulation by the arsenic presence could trigger the expression and activity of antioxidant enzymes. Our results show only a significant increase in catalase activity in response to As(V) (Figure 6B). As it was mentioned before, this is coherent with the redox imbalance caused by the As(V) reduction. Unlike As(III), that triggers a tolerance mechanism based only on expulsion. On the other hand, superoxide dismutase activity remained mostly unchanged for both arsenic conditions (Figure 6C). A small increase in SOD activity was only significant in SH1S21 strain against As(V).

### Arsenic Response Gene Expression

**FIGURE 7:**
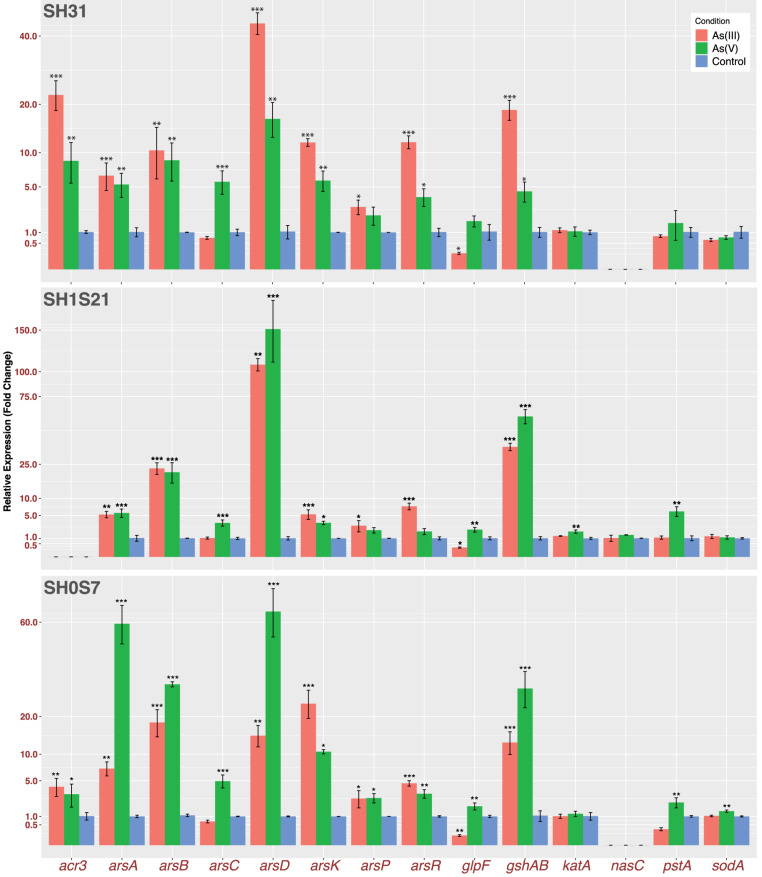
Transcriptomic expression of arsenic related genes. Bars represents relative fold change gene expression related to control condition of selected genes. Plotted data is an average of three independent experiments with three technical replicates each (*p ≤ 0.05; **p ≤ 0.01; ***p ≤ 0.001).

Our results show an active response to arsenic stress on the three strains, despite of different gene expression magnitudes among the strains. We measured genes representing the following functions: direct response to arsenic (*arsRDAB, arsC, arsP, arsK* and *acr3*); arsenic uptake (*glpF* and *pstA*); oxidative stress response (*gshAB, katA* and *sodA*) and finally, we also wanted to test if this nitrate reductase (*nasC*) could be responding to arsenic (Figure 7). As expected, the genes from the first group showed induction for both species of arsenic on the three strains. The only exceptions were *acr3* that is not present in the genome of strain SH1S21 and *arsC* that only have a role in the presence of As(V). As a consequence of the reduction, the *arsRDAB* operon, [which responds to As(III)], is also activated in the presence of As(V). On the other hand, *arsP* also appears to be responding, but in a smaller magnitude compared to the other transporters. It is worth mentioning that the As-methylase enzyme necessary for ArsP transporter activity is yet to be found in the *Exiguobacterium* genus.

With respect to arsenic importers, we measured the expression of *glpF* and *pstA* and found that there is a change in their regulation due to the arsenic presence, aiming to block the influx. Moreover, among the genes related to oxidative stress response, *gshAB* encodes for the main glutathione biosynthesis enzyme which is highly induced on the three strains in both arsenic conditions. This is consistent with an active response to face the arsenic induced oxidative stress. Although, there is no significant change in *katA* and *sodA* expression, that correlates with SOD enzymatic activity, but not with KatA activity that did show induction by As(V). Finally, the *nasC* expression (present in the SH1S21 strain) showed no significate change in response to any arsenic treatment. This could be supporting the assertion of this cluster working under anoxic conditions, in any case its specific role or function remains unknown.

## Discussion

Altiplanic environments have extreme and variating conditions, even between geographically close areas (Cabrol et al., 2009a; Hernández et al., 2016; Cortés-Albayay et al., 2019). Particularly, a previous work in SH showed highly different values of conductivity, dissolved nitrogen and dissolved organic carbon, between two ponds just a few meters apart (Aguilar et al., 2016). In our study, arsenic concentration and salinity are the two most prominent factors with the greatest variation (Figure 1). Moreover, salinity variation correlates with previous reports for H0, H1 and H4 sites (Dorador et al., 2008; Remonsellez et al., 2018), even they found that H0 and H1 sites exhibited more similarities, contrasting with the remaining sites. Hence, the effects of the five SH niche conditions (arsenic specifically), over the micro-diversity of inhabitant *Exiguobacterium* strains is of great interest.

The great variability in the water and sediment chemical composition is due to the salar hydrogeography (Acosta and Custodio, 2008). Season percolation mobilizes minerals and the high evaporation rate causes their concentration and stratification. Additionally, flow from the underground sources bring the minerals to the streams that feeds the water bodies (Hernández et al., 2016). This could explain the arsenic and salinity gradient from north to south (Castro-Severyn et al., 2019). Furthermore, biotic processes like metabolism and primary producers activity from the highly variable microbial communities, can also contribute with changes in water and sediment properties (Oren, 2013; Aguilar et al., 2016).

Our genomic results showed that phylogenetic relationships further confirm the segregation of this genus into two large groups (Figure 2 and Supplementary Figures S1–S3), organized according isolation environments in most cases (Konstantinidis and Tiedje, 2005; Richter and Rosselló-Móra, 2009). Hence, as the SH strains come from a common ancestor, we can suggest that their current observed diversity is product of evolutionary adaptation, caused by the separation of different lineages within the microenvironments of SH. Implying that the plasticity and divergence presented by strains of this genus could be due to shaping effect of key niche variables. On the other hand, we cannot discard the effect of mutations accumulation between strains that diverged from a common ancestor and do not exchange genetic material (Pérez-Losada et al., 2018).

Besides, here we found more evidence of the relatedness between the *E.* sp. S17 strain isolated from the Argentinian altiplano and the Huasco strains (Ordoñez et al., 2013; Castro-Severyn et al., 2017). Strikingly this is not the case for *E. chiriqhucha* N139 which was also isolated from the Argentinian altiplano (Gutiérrez-Preciado et al., 2017), it might be explained because this strain was isolated from the water column. Moreover, 67.6% of the analyzed genomes (50/74) are not classified according to species level, which also correspond with the great diversity within the *Exiguobacterium* genus.

Establishing the pan-genome of a bacterium sheds light on its biology, lifestyle and has implications for the species definition (Tettelin et al., 2008). The data reported previously seems to support the idea that *Exiguobacterium* genus has an open pan-genome, thus confirming high level of intra-species diversity (Castro-Severyn et al., 2017). Which is coherent with the small number of available sequenced genomes for this specie (77 until May 2019) and isolated of very diverse environments. In this work we focused on the degree of diversity that could exist between strains of different niches of the same environment (SH). Our results showed a large pan-genome size (4,648 genes) with respect to that of individual genomes (average of 2,947 genes) (Figure 3). The phylogenetic relatedness and the ANI values (∼96.9%) among the SH strains evidence a common origin, so they would have diverged to adapt to niche particular conditions. Therefore, the origin of the divergence processes for the *Exiguobacterium* strains could be an allopathic diversification caused by ecosystem fragmentation, forming different and relatively isolated niches in the SH (Rouli et al., 2015).

Altiplano environments fragmentation has been occurring at different time scales and by multiple phenomena. An important example that generates changes in geography is the water cycle or balance (“El Niño” phenomenon, amount of precipitation and the high evaporation rate in this area) during the climate change cycle (Placzek et al., 2006). Also, the great volcanic activity and tectonic movements in this area not only physically fragment the environments, but also generate changes in the flows and physicochemical properties of soils and waters, promoting heterogeneity (Risacher and Fritz, 2009). Even human activities, such as mining and water extraction from aquifers, cause a great impact, that has been demonstrated on a small scale within the Salar de Huasco (Acosta and Custodio, 2008).

The results lead us to suggest that these geographical and physicochemical changes that have occurred in the SH area since its origin are reflected by the organisms that inhabit it. Consequently, these were progressively being separated, isolated and faced with changing conditions, leading to the selection of those who managed to adapt. In this sense, open pan-genomes can be indicators of species that live in multiple environments and are part of highly variable microbial communities such as the ones found in SH (Dorador et al., 2010, 2013). Thus, increasing the probability of gene transfer phenomena between the community members and the pan-genome would keep growing (McInerney et al., 2017). This presents the opportunity for *Exiguobacterium* strains to obtain niche or community exclusive genes, contributing to their diversity and to the species pan-genome size.

As we stated that the strains could have isolated and diverged over time, we cannot rule out the effect of other forces which could be causing bacteria from diverse niches to interchange or mix. In fact, the effect of wind, water flow and even animals (e.g., Llamas), on microorganism’s mobilization and dissemination is very well documented (Augspurger et al., 2010; Smith et al., 2013; Alm et al., 2018). Although there is no information on this subject for the SH, there are studies describing flow models and ground water networks (Acosta and Custodio, 2008).

All these factors could explain or contribute to the discrepancy observed when contrasting the size and composition of the pan-genome with the phylogenetic positioning and average nucleotide identity among the SH strains. Nevertheless, the variability among the SH strains is indeed evidenced by the differential COG pattern of each strain accessory genome and the number of genes. We were expecting that the specific niche adaptations and differential physiological capacities among these strains emerged from the accessory genes. However, with our results we cannot clarify this, because most of our identified genes are mainly part of the core or disposable genome compartments and the majority of the accessory compartment genes are unclassified or with unknown function. Hence, to solve this, further analyzes are needed. Although, some approaches have currently been developed to solve this, as discussed in: da Costa et al., 2018; and Antczak et al., 2019. Briefly, they use combinations of many predictive tools to gather and add evidence, assigning possible functions with a good confidence level to those unclassified or unknown proteins.

In a broad sense, most of the studied markers (related to arsenic tolerance, oxidative and global stress) are present in all strains (Supplementary Figure S4). However, there is also a clear differential pattern between the strains of the three environments (SH, GSL and EI), which is consistent with their particular arsenic tolerance levels. For example, *uspA*, and several arsenic related ones like *arsR, arsC, arsP, arsK* and *acr3* (Figure 4). Although our results imply that the presence of arsenic response genes such as *arsC, acr3* and perhaps *arsK* and *arsP* indeed increases the resistance level (SH strains regarding those from GSl and EI), this does not explain its heterogeneity among the SH strains. Moreover, an explanation could be the regulation and expression impact on arsenic response, which has been discussed in other published works (Cleiss-Arnold et al., 2010; Castro-Severyn et al., 2019). Also, we support the participation of global and oxidative stress response systems role in strengthens this resistance capacity.

Moreover, enrichment in genes directly related to arsenic tolerance on SH strains is evidence of the selective pressure exerted by the presence of this toxic compound (Slyemi and Bonnefoy, 2012; Andres and Bertin, 2016). Particularly, *arsC, arsP* and *acr3* are only found in SH strains, additionally a second copy of *arsR* (to regulate *arsP*) is also present. However, *acr3* gene is missing in the three most sensitive SH strains (from H1 site), which could be evidence of the important role of ACR3 expelling function in the arsenic resistance, which has been reported before (Fu et al., 2009; Ordoñez et al., 2015). ArsP is known to be an organoarsenical permease (Shen et al., 2014), with a potential role in *Exiguobacterium* tolerance that had not previously been avowed. On the other hand, *arsK* gene is present in the GSL and most SH strains, but not in those from EI. Hence, resulting in the logical pattern observed, regarding the arsenic concentration between the three environments and the enrichment of genes displayed by these strains to face it. Since arsenic has been detected on the GSL basin in lower concentrations (0.4 – 95 μg/L for water) regarding the SH (Waddell, 2004; Adams et al., 2015). Conversely, there are no reports for arsenic in EI.

Taken together, most of SH strains have three clusters with arsenic response markers (*arsRDAB, arsP* and *arsK-acr3*), that could be under different regulations. Previous reports described strains with more than one arsenic gene cluster, which responds to different signals such as aerobia/anaerobia (Saltikov et al., 2005) or As concentration (Zhao et al., 2015). Since it is known that both ArsP and ArsK expel organic arsenics, it is necessary to find the missing piece (As-methylase enzyme) to promote this as a functional mechanism in *Exiguobacterium* (Garbinski et al., 2019). On the other hand, ArsK can also expel As (III), so its function could be complementing/increasing the bacteria resistance. Nonetheless, these genes gave us some hints supporting the idea of a more varied repertoire that yields higher resistance. However, we have to consider that *arsK* is present in the GSL strains which were the most susceptible ones, so the role of this gene remains unclear.

Another interesting feature found in some of the studied genomes was the nitrate reductase gene cluster. Although being exclusively present in some SH strains, its distribution does not present a pattern of association by site or arsenic tolerance level, but it does show a clade specific pattern. On the other hand, is important to take into consideration, that there could be a selection pressure that we are not considering, which pushes *Exiguobacterium* genomes toward convergence. However, we could not conclude that its presence has any relation with arsenic or environmental stress adaptation.

In addition, a poorly characterized anaerobic arsenite oxidase (*arxA;* molybdopterin containing enzyme) was identified in the genome of a Mono Lake (California, United States) isolated that couples arsenite oxidation with nitrate reduction (Zargar et al., 2010). Furthermore, we were not able to detect an *arxA* sequence in any of the studied *Exiguobacterium* genomes, we cannot rule out the presence or function of some analog. But we did find within this cluster an uncharacterized 2Fe-2S protein that shares some characteristics with arsenite oxidases. As we know the effects of lacking information and databases availability for the description of new mechanisms, we believe that this could be re-addressed when more and new information is generated.

Another finding that supports that a novel mechanism may be in play is the presence of eleven genes related to the molybdopterin biosynthesis are in this same genomic context, which is a well-known nitrate reductase co-factor (Kelley, 2017). Also, this molecule is a co-factor for ArxA too (Zargar et al., 2010). Besides, the presence of the transcriptional fumarate and nitrate reductase regulator *fnr* in this genetic cluster could be evidence of an anoxic related response (Osorio et al., 2019). Finally, as most of our studied markers are part of the core genome compartment, we would imply that differences in regulation and expression levels among the strains could be the source of their variable arsenic tolerance levels. On the other hand, we have to consider that these determining factors may be among the hypothetical and unknown function markers of the accessory genomes. Also, all these results further support the plasticity of the *Exiguobacterium* genus and suggest that environmental factors of each niche could shape the species by driving divergence.

Oxidative stress is an important arsenic toxicity mechanism and detoxifying enzymes has always been a target of interest (Harrison et al., 2009). Increase in catalases and superoxide dismutases expression and activity in response to arsenic, as well as the loss of this tolerance due to mutations in these enzymes are evidence of their role (Parvatiyar et al., 2005). The results show that both arsenic tested conditions were able to promote intracellular ROS accumulation in the analyzed strains (Figure 6). Which could be counteracted by catalase and superoxide dismutase activity. Detoxification of As(V) comprises an oxidation-reduction process that contributes to oxidative stress by depleting the thiols altering the cell redox balance (Mukhopadhyay et al., 2002; Daware et al., 2012). Generally, As(III) was not able to generate a significant increase of enzymatic activity on the three strains. Moreover, the lower increase in SOD activity was an unexpected result, since it has been reported that these enzymes respond to arsenic in *H. arsenicoxydans* and *K. pneumoniae* (Cleiss-Arnold et al., 2010; Daware et al., 2012), suggesting that they might be using another detoxifying mechanism. Nonetheless, this may be because As(III) induces less ROS formation and/or accumulation which is effectively counteracted by the present enzymes (Mateos et al., 2017). Oxidative stress resistance could cause tolerance against other stresses, as was demonstrated in *Deinoccocus radiodurans*, in which high UV stress resistance is associated with its SOD efficiency (Markillie et al., 1999).

Most of the arsenic related genes showed a significative upregulation in response to both arsenic species, on the three strains (Figure 7). Moreover, all transporters showed different induction levels, supporting the toxic expulsion as the main resistance mechanism used by these bacteria. In addition, another strategy used by bacteria to tolerate the arsenic present in their environments is to block the influx, which correspond with *glpF* repression by As(III) (Elias et al., 2012). On the other hand, the genes that code for catalase and superoxide dismutase did not show significant expression changes in response to arsenic among the three strains. Whereas, glutathione synthesis appears to be highly active due to *gshAB* significate overexpression, thus helping to restore cell redox balance. Finally, we think that differential arsenic tolerance presented by these strains is the result or sum of a set of factors, markers and particular regulation, combining permeability, detoxification and homeostasis changes to ensure survival, as we described previously (Castro-Severyn et al., 2019).

**FIGURE 8:**
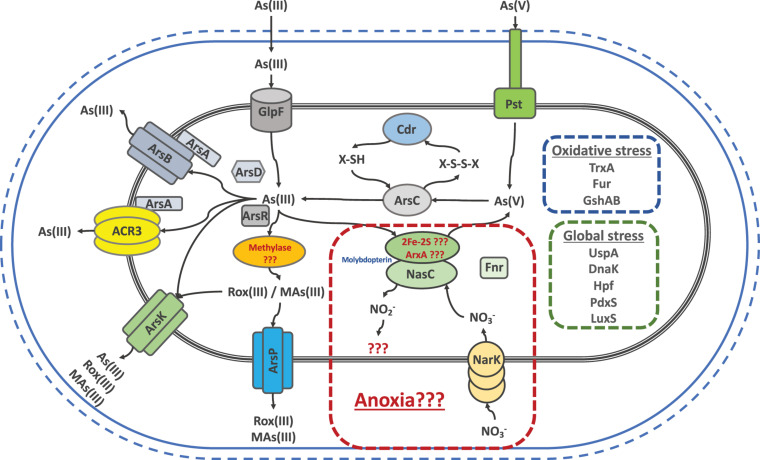
Schematic representation of genes and/or proteins that exist in the different Salar de Huasco isolated *Exiguobacterium* strains as a repertoire to respond against arsenic induced stress, as well as the consequent oxidative and global stresses.

To sum up, the joint efforts and results of all our work led us to state that the main strategy used by the SH *Exiguobacterium* strains against arsenic is based on the toxic expulsion out of the cell. To this end, these strains have a wide variety of efflux proteins like ArsB, ArsP, ArsK and ACR3 to cope with any arsenic species, since arsenic could be reduced, oxidized or methylated. Figure 8 shows a graphic summary which integrates our genomic, gene expression, proteomic and physiological findings (This work; Castro-Severyn et al., 2017, 2019). Arsenic toxicity to the cell is known to generate oxidative and global stress states. In order to fight this, several responses are activated, particularly against oxidative stress, Cdr could be restoring the thiols depleted during As(V) reduction. Furthermore, PdxS which is known to be involved in several types of stress resistance, is also a cysteine synthesis cofactor, aminoacid needed by the GshAB to form new thiols, counteracting the oxidative stress along with all of TrxA copies. Also, our results support all the reported evidence promoting global stress markers such as LuxS, Hpf, DnaK and UspA as key players in arsenic resistance. Finally, we can suggest that the presence of ACR3 increases the level of resistance to the strains that possess it, such as the SH ones. However, the variability between the levels of these same strains may be due to the addition of other factors such as ArsK, ArsP and the global and oxidative stress markers and its regulation, that would be contributing to the resistant phenotype.

## Conclusion

Salar de Huasco, as part of the Chilean altiplano, is indeed a very extreme and diverse environment in which arsenic appears to be one of the main communities shaping factors. As a whole, these results indicate that there are key elements at genome level that enable those bacteria to respond poly-stress and in particular As presence. Additionally, phylogenetic relationships and pan-genome composition found among the *Exiguobacterium* strains could imply that these adaptations emerge from their particular niche. These observations are consistent with the transcriptional and enzymatic responses against arsenic of these bacteria. In this sense, the strategies used by the arsenic tolerant/resistant bacteria and/or communities should be studied with a multidisciplinary vision to obtain a better understanding of the occurring phenomena, interconnecting results to establish correlations applying an ecological perspective to particular events. Finally, the increase in database information and the use of omics approaches is currently generating more data that is shedding light in understanding the evolutionary process of adaptation, especially on extreme environments.

## Data Availability Statement

The genomic data presented in this study has been deposited in the DDBJ/ENA/GenBank under the BioProject: PRJNA319980.

## Author Contributions

JC-S, EC-N, and CS conceived and designed the study. JC-S, FM, FR, and CS performed the field work. JC-S, CP-E, and NM performed the experimental procedures. JC-S, KM, SM, and EC-N analyzed genomic data. CS, FR, and EC-N contributed with reagents, materials, and analysis tools. JC-S, CP-E, EC-N, FR, and CS wrote the manuscript. All authors read and approved the final manuscript.

## Conflict of Interest

The authors declare that the research was conducted in the absence of any commercial or financial relationships that could be construed as a potential conflict of interest.

## Footnotes

1 http://tree.bio.ed.ac.uk/software/figtree/

2 https://github.com/dparks1134/CompareM
