# Supplementary material for: Genomic Variation and Arsenic Tolerance Emerged as Niche Specific Adaptations by Different Exiguobacterium Strains Isolated From the Extreme Salar de Huasco Environment in Chilean – Altiplano
Source: Front Microbiol. 2020 Jul 15;11:1632. doi: 10.3389/fmicb.2020.01632 (PMC7374977; doi:10.3389/fmicb.2020.01632)
Supplement: Supplementary file 4 [file Data_Sheet_1.PDF]

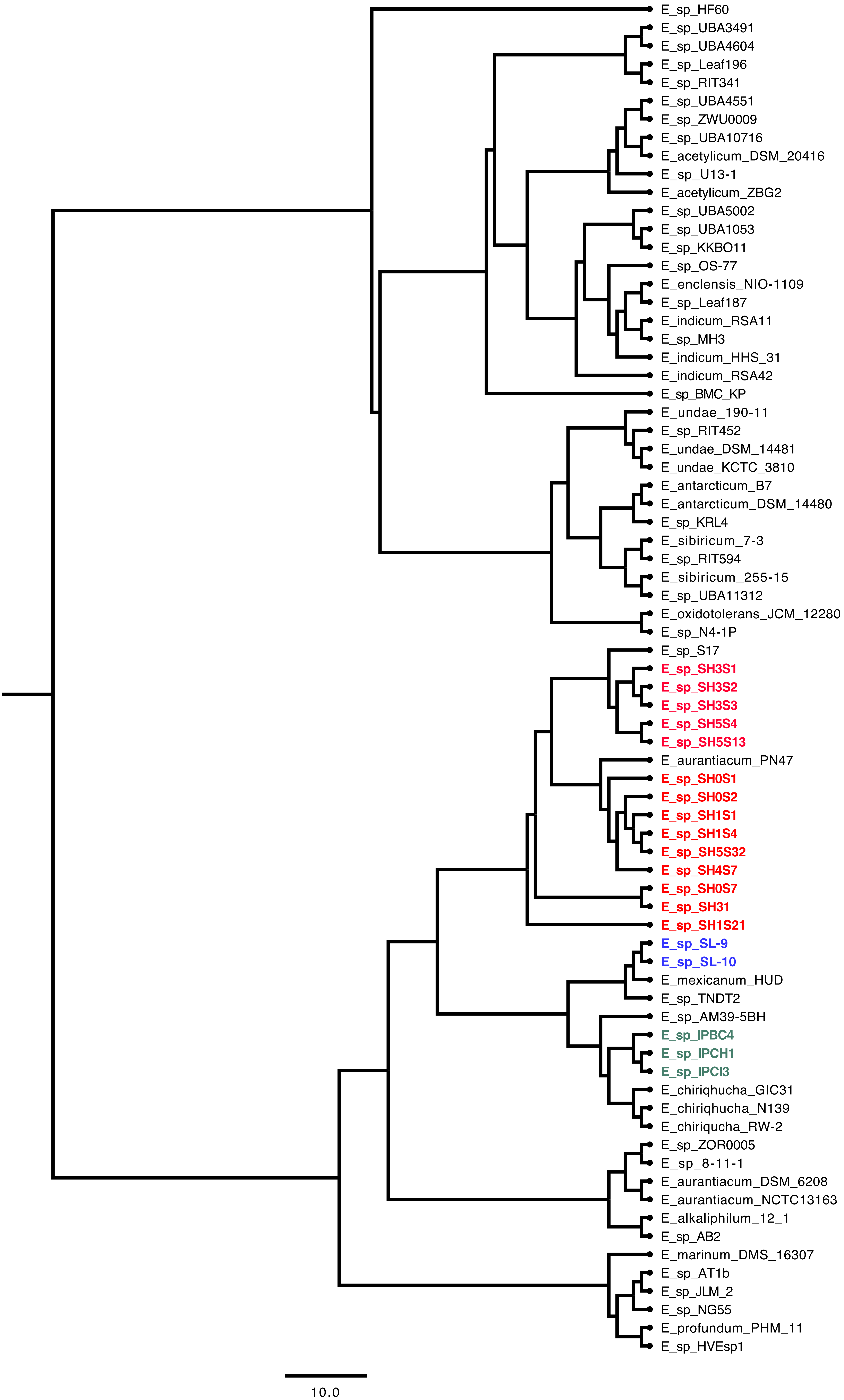

Supplementary Figure S1. Filogenic reconstruction of the *Exiguobacterium* genus. Mid-point rooted Phylogenetic tree inferred from an alignment of 31 conserved genes of our 19 strains plus other 55 available in the GenBank.
