# Supplementary material for: Genomic Variation and Arsenic Tolerance Emerged as Niche Specific Adaptations by Different Exiguobacterium Strains Isolated From the Extreme Salar de Huasco Environment in Chilean – Altiplano
Source: Front Microbiol. 2020 Jul 15;11:1632. doi: 10.3389/fmicb.2020.01632 (PMC7374977; doi:10.3389/fmicb.2020.01632)
Supplement: Supplementary file 6 [file Data_Sheet_3.PDF]

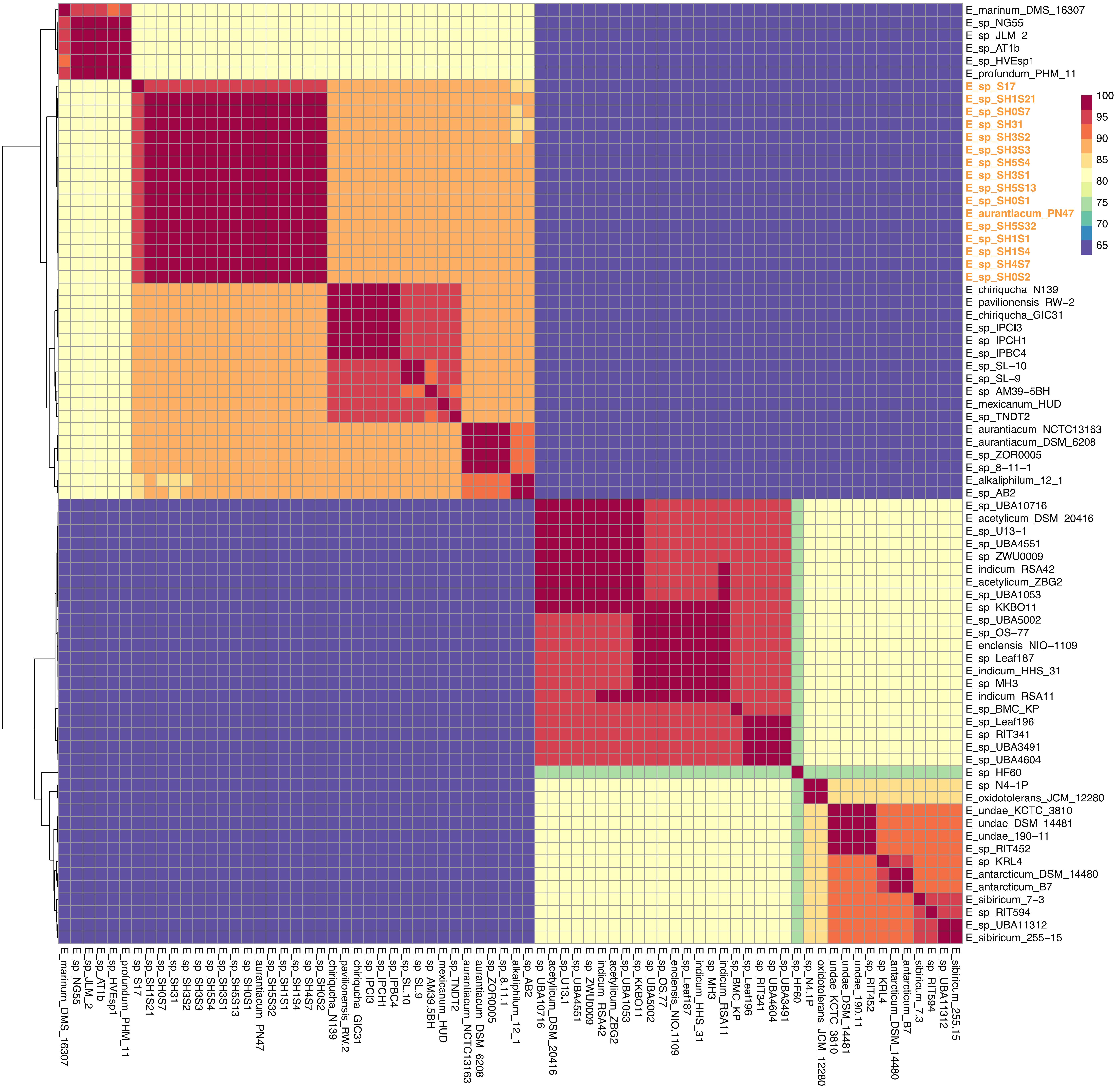

Supplementary Figure S3. Average amino-acid Identity of the *Exiguobacterium* genus. Pairwise comparison of our 19 strainsplus other 55 available in the GenBank. Hierarchical clustering of the based on their average identity values. The color gradients show the percentage of identity, from lowest to highest, that each pair of genomes shares.
