# Supplementary material for: Genomic Variation and Arsenic Tolerance Emerged as Niche Specific Adaptations by Different Exiguobacterium Strains Isolated From the Extreme Salar de Huasco Environment in Chilean – Altiplano
Source: Front Microbiol. 2020 Jul 15;11:1632. doi: 10.3389/fmicb.2020.01632 (PMC7374977; doi:10.3389/fmicb.2020.01632)
Supplement: Supplementary file 7 [file Data_Sheet_4.PDF]

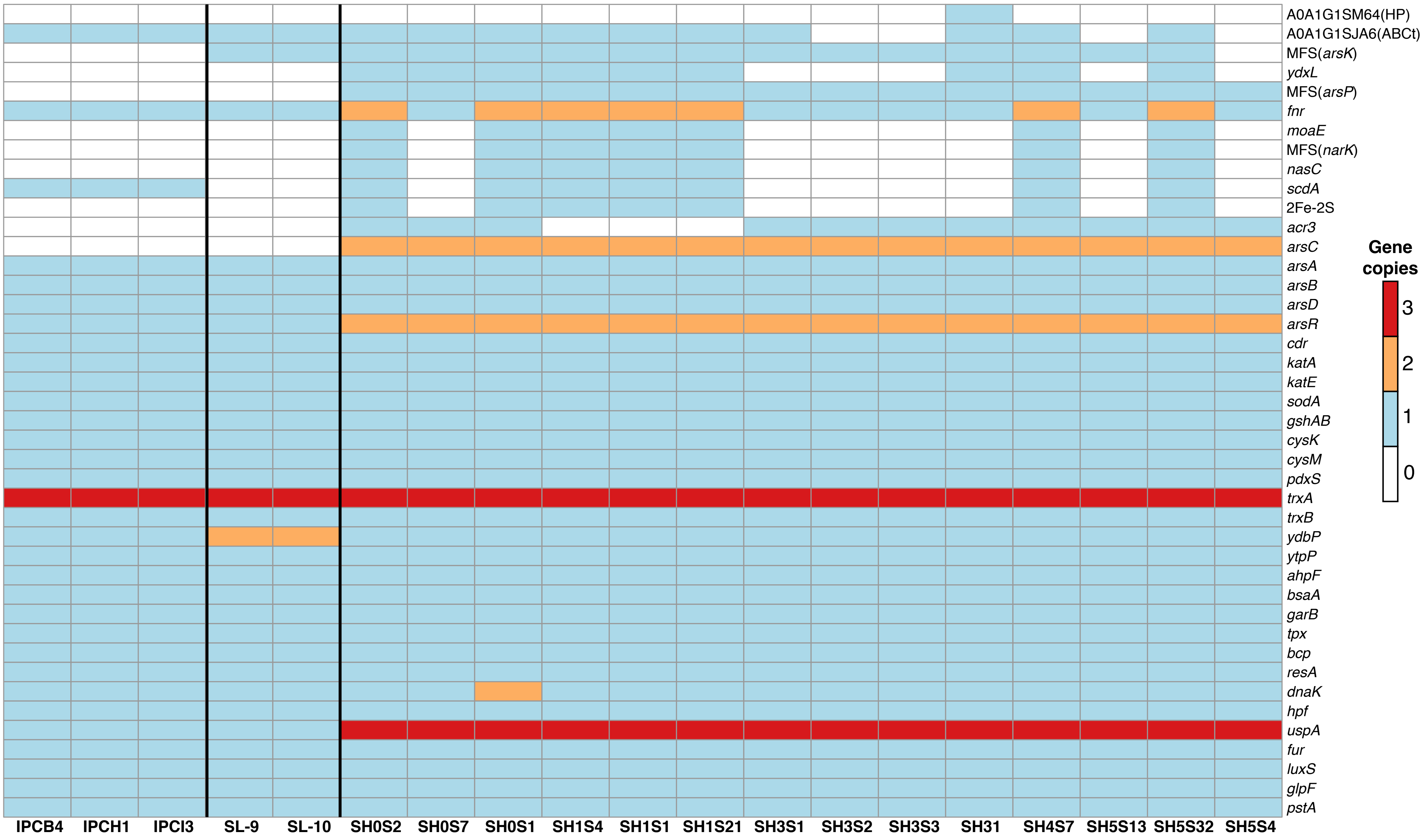

Supplementary Figure S4. Distribution and copy number of stress-related genes among the *Exiguobacterium* genomes. Genes are color-coded by copy number.
