# Supplementary material for: Genomic Variation and Arsenic Tolerance Emerged as Niche Specific Adaptations by Different Exiguobacterium Strains Isolated From the Extreme Salar de Huasco Environment in Chilean – Altiplano
Source: Front Microbiol. 2020 Jul 15;11:1632. doi: 10.3389/fmicb.2020.01632 (PMC7374977; doi:10.3389/fmicb.2020.01632)
Supplement: Supplementary file 8 [file Data_Sheet_5.PDF]

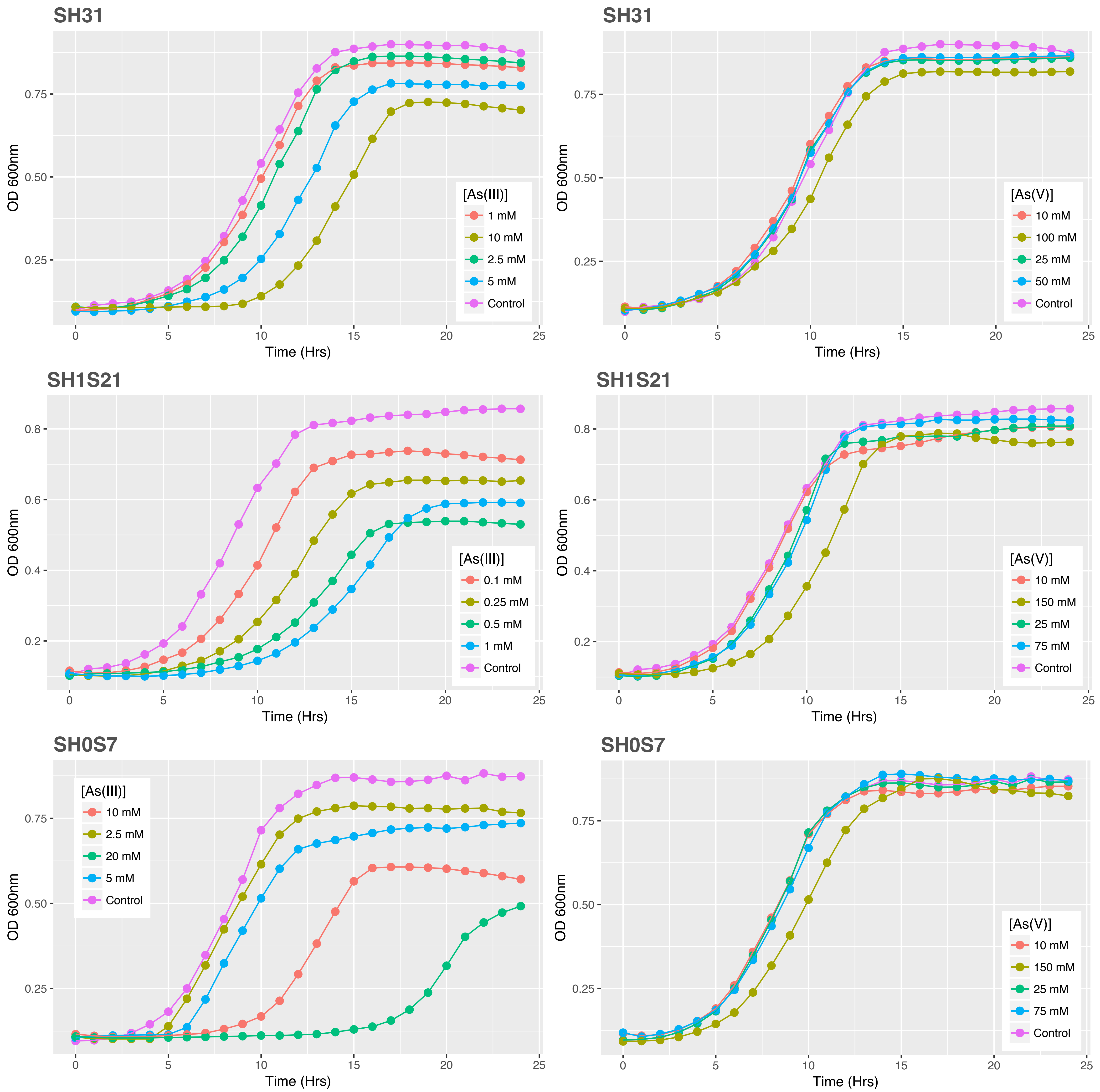

Supplementary Figure S5. Growth curves of the three studied strains, under the tested conditions: control, As(III) and As(V) at different concentration depending of the strains. OD600 readings were recorded during 24 h. Mean values (n = 3) are plotted.
